# Supplementary material for: Ketamine Increases Proliferation of Human iPSC-Derived Neuronal Progenitor Cells via Insulin-Like Growth Factor 2 and Independent of the NMDA Receptor
Source: Cells. 2019 Sep 24;8(10):1139. doi: 10.3390/cells8101139 (PMC6830315; doi:10.3390/cells8101139)
Supplement: Supplementary file 1 [file cells-08-01139-s001.pdf]

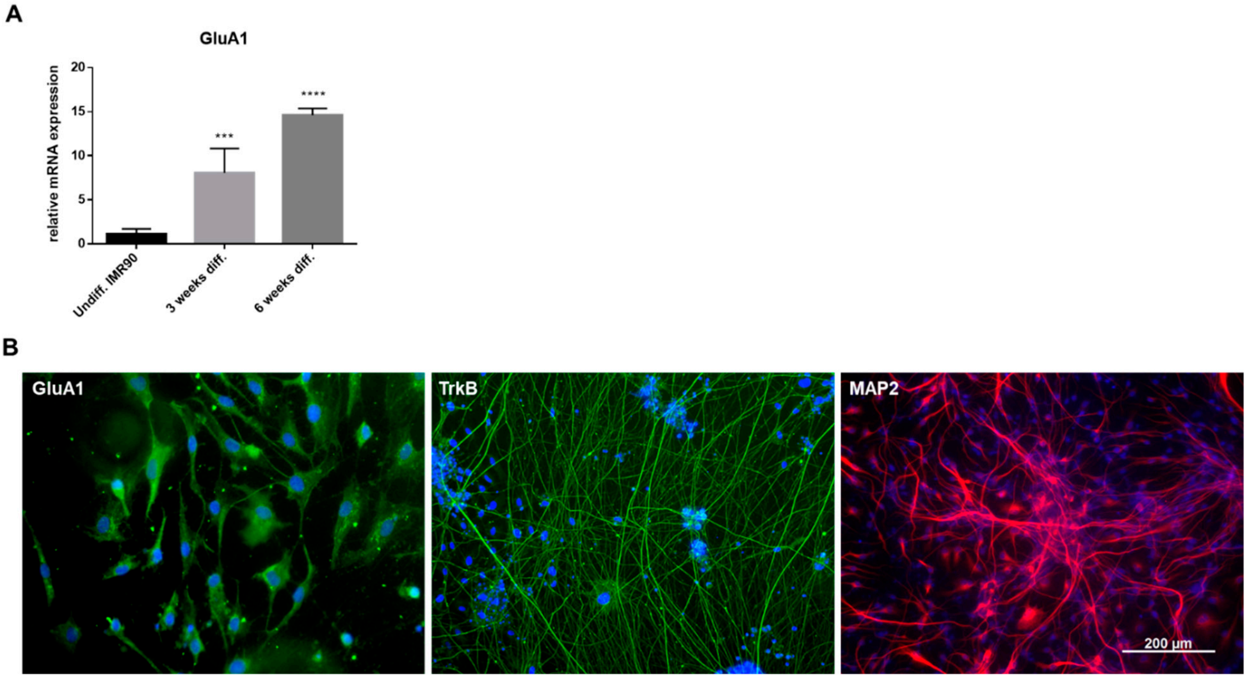

Figure S1. Characterization of differentiated IMR90 neurons. (**A**) qPCR showing upregulation of AMPA receptor subunit GluA1 after 3 and 6 weeks of differentiation. Differentiation of IMR90 NPCs was performed in poly-L-ornithine/laminin coated black 96-well clear bottom plates. Differentiation medium (BrainPhys medium (stemcell technologies), 1x B27 without vitamin A (Invitrogen), 1x N2 (Invitrogen), 20 ng/ml GDNF (Peprotech), 20 ng/ml BDNF (Peprotech), 20 ng/ml NT3 (Peprotech), 1 mM cAMP (Sigma Aldrich), 0.2 µM Ascorbic acid (Sigma Aldrich)) was changed every other day for 6 weeks. (B) Immunocytochemical characterization of differentiated human iPSC-derived neurons after 6 weeks showing protein expression of ionotropic glutamate receptor AMPA-R (GluA1 subunit), brain-derived neurotrophic factor (BDNF) receptor TrkB and microtubule associated protein 2 (MAP2), scale = 200 µm.

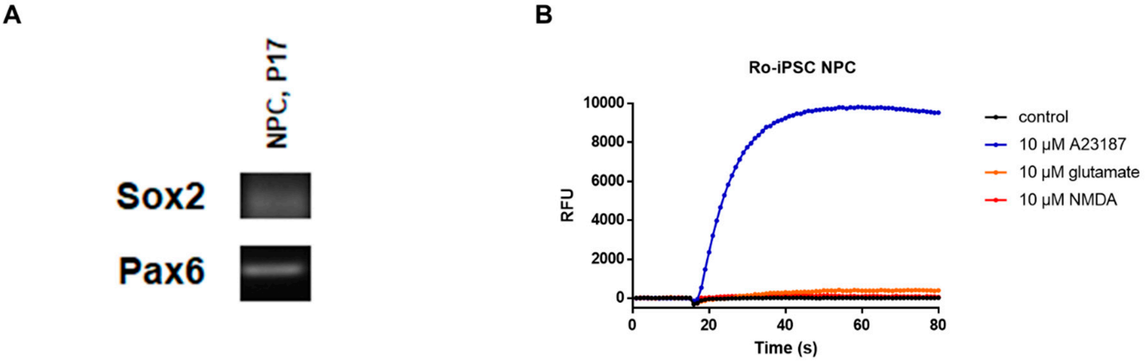

Figure S2. Characterization of Ro-iPSC NPCs. (A) RT-PCR analysis of the neuronal progenitor markers Sox2 and Pax6 mRNA in Ro-iPSC NPCs (passage 17). The following primers were used: F-hPax6: AATAACCTGCCTATGCAACCC, R-hPax6: AACTTGAACTGGAACTGACACAC (214bp); Figure 2. ATGCACCGCTACGACGTGA, R-hSox2: CTTTTGCACCCCTCCCATTT (437 bp). (B) Functional analysis of NMDA-receptors in two human iPSC-derived Ro-iPSC NPCs using Fluo-8 Calcium rise assay. Cells were stimulated with agonists for the NMDA-receptor, (NMDA and glutamate) and calcium rise was measured. Upon stimulation with NMDA (10 µM) or glutamate (10 µM), no increase of calcium accumulation was observed in NPCs. The calcium ionophore A23187 (10 µM) generated the maximum fluorescence signal and served as a positive control.

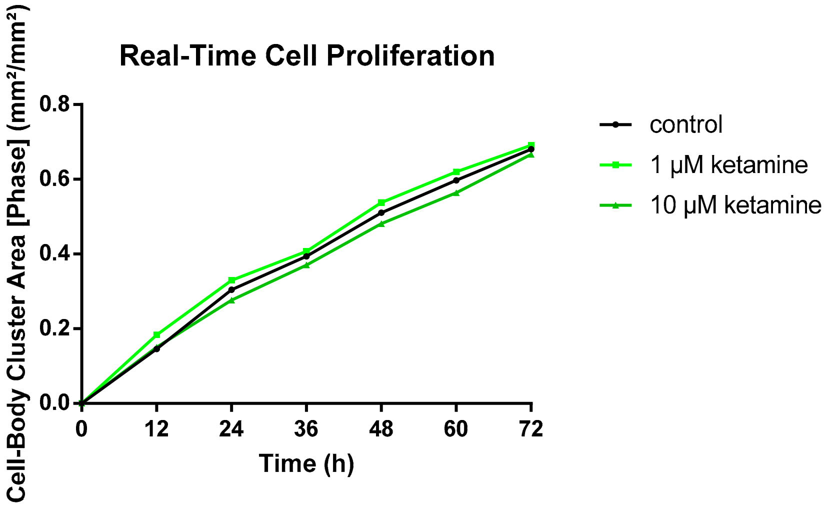

Figure 3. Ketamine showed no effect on proliferation of human cervix epithelioid carcinoma cells ("HeLa cells"). Phase-contrast imaging was performed using the IncuCyte® Zoom time-lapse microscopy system at 37 °C for a period of 72 h. NPCs were treated with either 1 µM and 10 µM ketamine or 0.01% DMSO control. Confluency of cells was determined with IncuCyte® NeuroTrack Software indicated as Cell-Body Cluster Area.

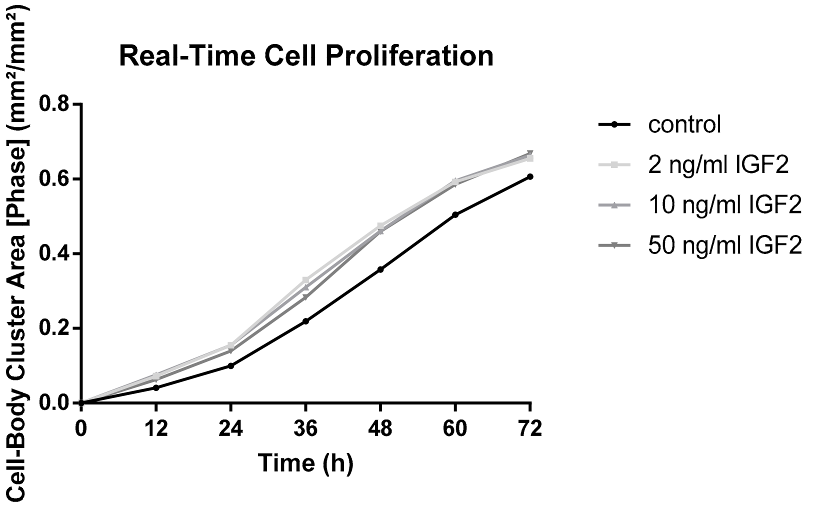

Figure 4. Treatment with IGF2 induced slight proliferation of IMR90 NPCs in insulin containing (20 µg/ml) maintenance medium**.** Phase-contrast imaging was performed using the IncuCyte® Zoom time-lapse microscopy system at 37 °C for a period of 72 h. NPCs were treated with either IGF2 (2, 10 and 50 ng/ml) or 0.01% DMSO control. Confluency of cells was determined with IncuCyte® NeuroTrack Software indicated as Cell-Body Cluster Area.

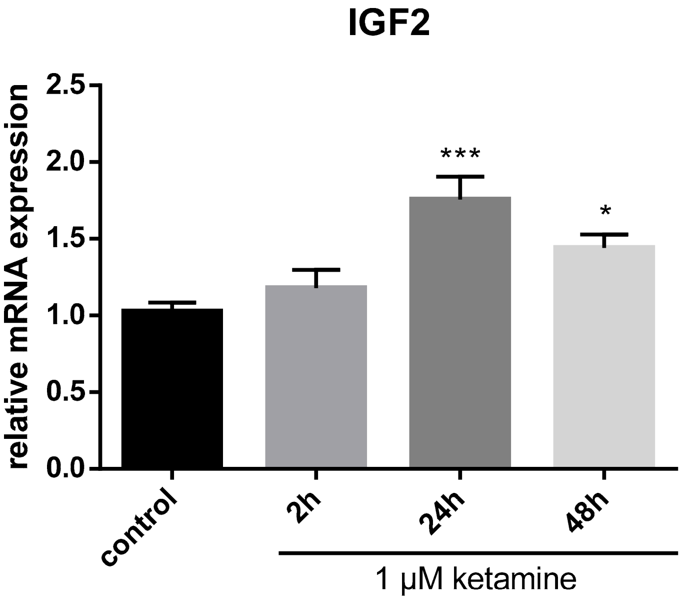

Figure 5. Time course of IGF2 mRNA expression. IMR90 NPCs were treated with 1 µM ketamine for the time points indicated. Ketamine increased IGF2 mRNA expression in a time-dependent manner and induced maximum upregulation after 24 h of treatment. The data represent means of three independent experiments. Error bars were calculated using ±SEM. p-values were calculated against DMSO control using one-way ANOVA and the underlying Sidak's multiple comparisons test.
